# Factor XIII Deficiency: A Silent Bleeder Behind a Normal Coagulation Profile

**DOI:** 10.1002/ccr3.72193

**Published:** 2026-03-06

**Authors:** Ramesh Yadav, Madhav Raj Karki, Dikshya Pokhrel, Aron Neupane, Kshitiz Parajuli, Hem Sagar Rimal

**Affiliations:** ^1^ Department of Paediatrics Birat Medical College Teaching Hospital Morang Nepal; ^2^ Birat Medical College Teaching Hospital Morang Nepal

**Keywords:** cranial, epidural, factor XIII deficiency, hematoma, umbilicus

## Abstract

Factor XIII deficiency is a rare cause of bleeding disorder. An 8‐month‐old male infant presented with persistent bleeding from a minor lip cut injury, which was sustained 2 days prior. The bleeding did not stop despite local hemostatic measures. His medical history was notable for spontaneous intradermal bleeds at different times and umbilical stump bleeding during the neonatal period. On examination, there were multiple petechiae and a large ecchymosis over the chest. Routine hematological and coagulation parameters were within normal limits. von Willebrand factor, factor VIII, and factor IX levels were normal. However, a qualitative 5M urea clot solubility test followed by factor XIII assay yielded abnormal results, confirming factor XIII deficiency. The patient was managed with fresh frozen plasma (FFP) and supportive care. Four months later, the patient presented with lethargy and vomiting, later diagnosed as epidural hematoma. The patient received FFP, had a favorable stay in the pediatric intensive care unit, and was discharged with no neurological deficits and a prophylactic plan. This case underscores how factor XIII deficiency can elude detection in routine coagulation tests, emphasizing the importance of prompt diagnosis and timely administration of replacement therapy to prevent complications and associated constraints in a resource limited setting.

## Key Clinical Message

Factor XIII deficiency can elude detection in routine coagulation tests in developing countries, emphasizing the importance of prompt diagnosis and timely administration of replacement therapy to prevent complications.

## 1 Introduction

Congenital bleeding disorders in infancy present a diagnostic challenge, especially when standard coagulation parameters are within normal limits. Inherited factor XIII deficiency (FXIIID) is a bleeding disorder that affects the final stage of the coagulation cascade system resulting in bleeding problems.

It can be inherited or acquired, inherited being autosomal recessive. The prevalence is one per 1–3 million people [1].

Because prothrombin time (PT), activated partial thromboplastin time (aPTT), and platelet counts usually stay normal, factor XIII deficiency is frequently missed by conventional coagulation screening, in contrast to other more prevalent coagulopathies. Clinical signs and symptoms can include life‐threatening cerebral bleeding, delayed wound healing, spontaneous soft tissue hemorrhages, and umbilical stump bleeding in neonates. This case highlights the importance of maintaining a high suspicion index for rare bleeding disorders in infants who present with unusual or prolonged bleeding, even in the presence of normal routine coagulation tests.

## 2 Case Presentation

### 2.1 Case History/Examination

An 8‐month‐old male infant presented to the emergency department with bleeding from a trivial cut on the upper lip following a fall from bed 2 days ago. Bleeding, however, was first noticed by the patient's parents only after 14 h. Medical attention was sought from a health assistant who packed the injury with gauze, but hemostasis was not achieved and referral to our hospital was made.

There was no history of fever, joint pain or swelling, rash, and blood in stool or urine.

**FIGURE 1:**
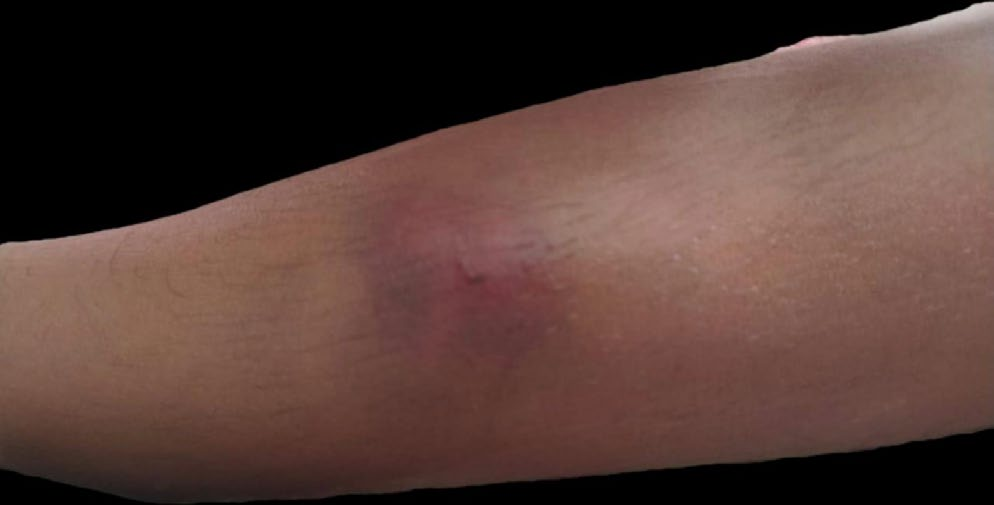
Superficial ecchymosis over the dorsum of the lower limb at a site of minimal trauma.

On examination, the patient's vitals were within normal limits. The patient did not show signs of blood loss. The upper lip had a cut injury of size 1 cm on the buccal surface with active bleeding. Physical examination revealed a single ecchymosis on the dorsum of the lower limb measuring 4 × 5 cm in size, along with numerous petechiae on the central chest, which were greenish to yellowish in color (Figure 1). Pallor was absent. Lymph nodes were non‐palpable, and there was no evidence of organomegaly. On interrogating the mother, a history of umbilical cord stump bleeding at 6 days of life and receiving blood transfusion for the same was obtained. A history of spontaneous intradermal bleeds since 6 months of age was retrieved in different parts of the body at various times. The patient's parents also reported prolonged bleeding with minor injuries for which no medical attention was sought due to geographical limitations.

He does not have any siblings. His parents had a non‐consanguineous marriage and no family history of bleeding disorders was retrieved.

## 3 Methods (Differential Diagnosis, Investigations, and Treatment)

The presentation raised suspicion of congenital bleeding disorders, consideration including hemophilia A or B, von Willebrand disease, platelet function disorders, and congenital factor deficiencies. Hematological examination revealed no abnormalities (Table 1). Additionally, blood sugar, liver function tests, renal function tests, and urinalysis were within normal limits. Tests revealed von Willebrand factor at 74.6%, factor VIII at 60.2%, and factor IX at 55%, all of which fell within the normal biological reference range.

**TABLE 1:** Hematological lab parameters of the infant.

Category	Investigation	Patient result
Complete blood count	Hemoglobin (Hb)	10.2 g/dL
	RBC count	3.7 × 10^12^/L
	WBC count	13,000/cmm
	Neutrophils	66%
	Lymphocytes	34%
	Eosinophils	0%
Coagulation profile	PT	13.4
	INR	1
	aPTT	33 s
	Bleeding time	3 min
	Clotting time	6 min 30 s
	Platelet count	258,000/mm^3^

Following these normal findings, we suspected factor XIII deficiency. A qualitative 5M urea solubility test was performed which was abnormal. As the patient had a unique history of bleeding from a healthy umbilical cord, we sent for FXIII activity assay to a lab in India. This later confirmed the diagnosis, as the test was unavailable in our hospital. Genetic analysis was not feasible due to financial constraints. The parents did not give consent to be tested for the same.

The patient was admitted to the pediatric ward and managed with a pint of fresh frozen plasma (FFP). Following administration, the bleeding from the cut injury ceased and the patient showed improvement. He was discharged and a follow‐up after 2 weeks was scheduled.

## 4 Results and Conclusion (Outcome and Follow‐Up)

**FIGURE 2:**
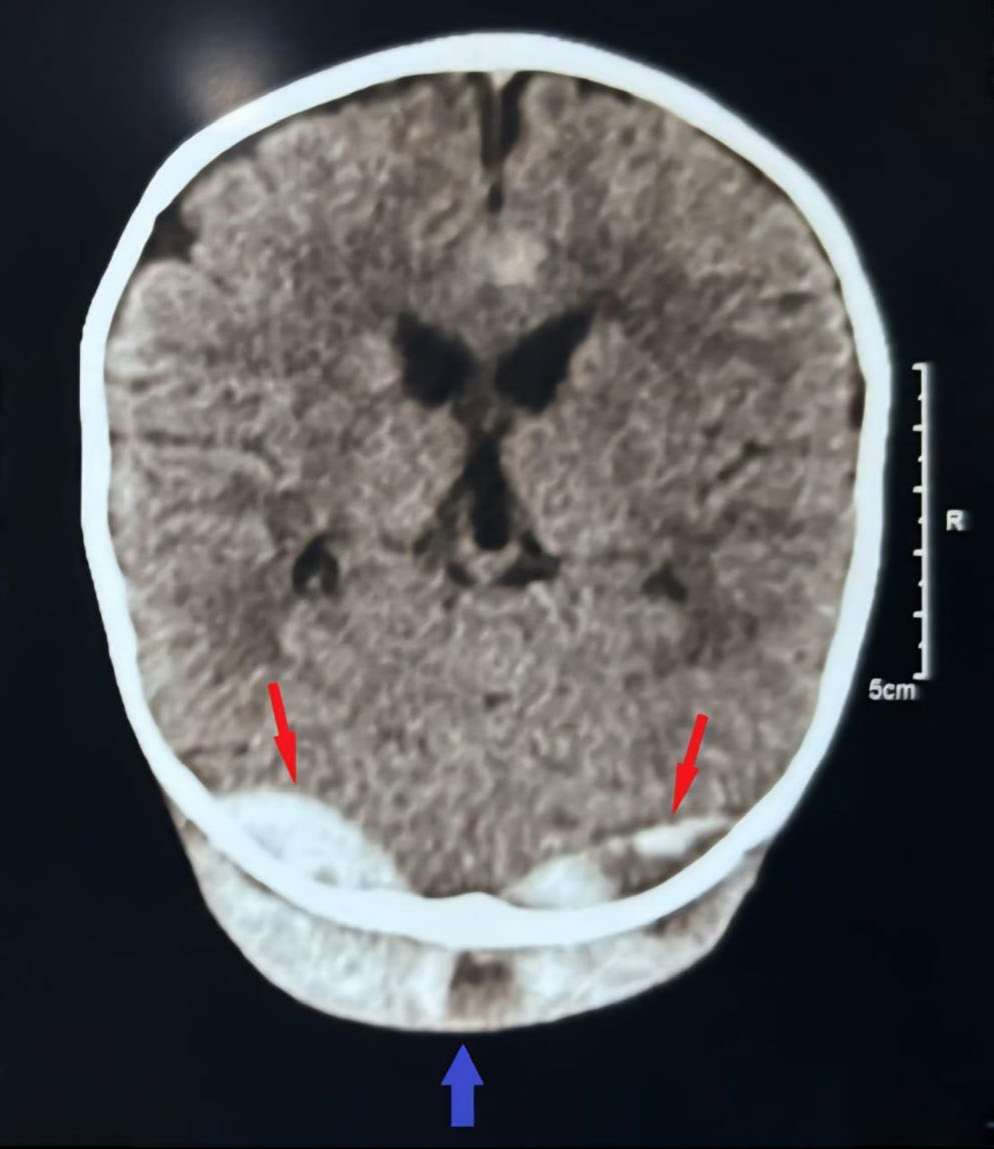
Crescentic shaped hyperdensity of blood attenuation are noted in both sides of occipital regions (red arrows). No perilesional edema, mass effect, midline shift, or fracture of overlying occipital bone is noted. Scalp swelling with soft tissue density within is noted in occipital region (blue arrow)—suggesting scalp hematoma.

The patient presented 4 months later with lethargy and an episode of vomiting following head trauma with impact over occipital region while playing. On examination, there was a localized swelling of size 2 × 2 cm in the occipital area. Non‐contrast cranial computed tomography showed epidural hematoma involving bilateral occipital regions (Figure 2). The patient was admitted to the pediatric intensive care unit, and a neurological consult was sought. He received 1 pint of FFP and was managed with supportive treatment. The patient remained stable and subsequent neurological examinations were normal. Following discharge, the patient was advised to follow up in the OPD at regular intervals including specialized oncology review.

The family was advised to monitor the child closely as part of their home care routine. Prophylactic FFP treatment every 1 month was advised thereupon after discussing the condition with the parents.

**FIGURE 3:**
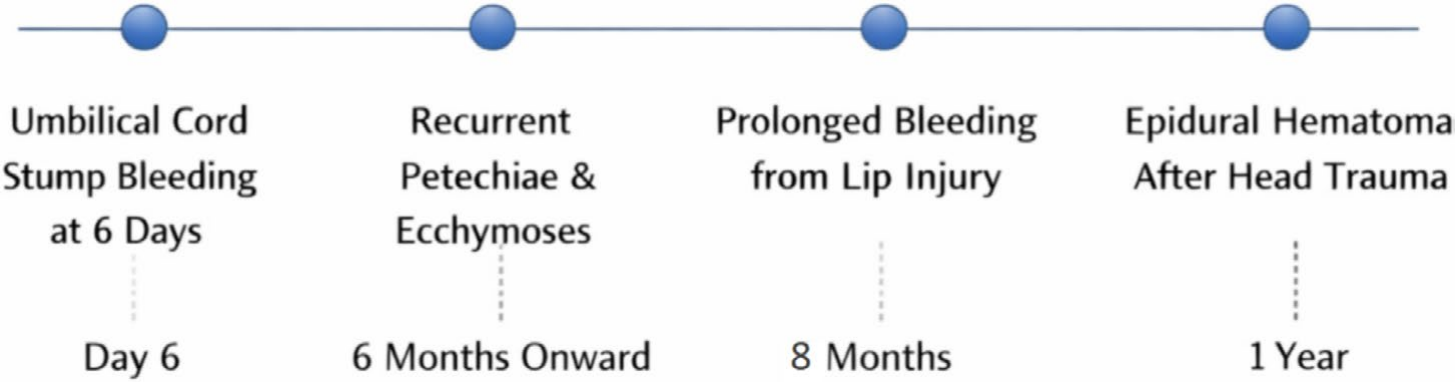
Timeline of clinical presentation.

The patient followed up after 2 months for prophylactic FFP and remained asymptomatic since the previous visit (Figure 3).

## 5 Discussion

Clotting factors are proteins required for blood clot formation. Among these, factor XIII plays a significant role in stabilizing the clot along with cross‐linking the fibrin polymer resulting in hemostasis. Factor XIII is made up of A and B subunits. The A subunit has catalytic activity, whereas the B subunit acts as the carrier. When activated, this factor cross‐links alpha and gamma chains, resulting not only in clot stability but also increases resistance to fibrinolysis and proteolysis during the last stage of the coagulation cascade [2].

Besides blood clotting and hemostasis, this clotting factor aids in wound healing, tissue healing, extracellular matrix formation, osteoblast differentiation, and modulation of immune responses at both the cellular and humoral levels. So, a deficiency of the factor results in a wide range of systemic effects ranging from adverse hemostatic consequences to dysregulated immune response. Factor XIII deficiency has both acquired and congenital forms. The acquired form mainly results from hyperconsumption, hemodilution, decreased synthesis or may be from production of antibodies targeting oneself. The majority of this variant is in the F13a1 gene located on chromosome arm 6p24‐25, encoding the A subunit. The acquired form is much more common than the congenital one. Families with traditions of consanguineous marriages have a greater incidence [3, 4]. Genetic counseling is necessary in such cases so as to prevent future pregnancy complications.

The clinical manifestations of the clotting factor deficiency are typical, which can become apparent at any age, but most patients are diagnosed during infancy when bleeding from the healthy umbilical cord remnant occurs. Symptoms often comprise persistent nosebleeds, bleeding from the gums, skin discoloration due to blood pooling under the skin (ecchymoses), firm masses of clotted blood (hematomas), extensive spontaneous bruising, and intracranial hemorrhage. The risk and prevalence of intracranial hemorrhage are greater in factor XIII deficiency than in any other inherited bleeding disorder [5, 6].

In case of trauma, bleeding symptoms occur after an hour or days, and intracranial bleeding is the most threatening and devastating complication [4]. Epidural hematoma is a rare presentation in factor XIII deficiency with one case showing a midline shift eventually requiring a left parietal craniotomy and other, a chronic right parietal epidural hematoma beneath the craniotomy flap from a previous emergent parietooccipital craniotomy and evacuation of the subdural hematoma. The previous case had an unfavorable course. It could not be identified as traumatic or spontaneous nontraumatic epidural hematoma in our case due to unremarkable history [7, 8].

Diagnosing factor XIII deficiency is challenging due to the disorder's infrequency and the fact that standard coagulation screening tests, including PT, aPTT, thrombin time, platelet count, and bleeding time, yield normal results. The diagnosis follows a sequential method, integrating family history, individual reactions to hemostatic challenges, and targeted laboratory assessments. Diagnosis of specific factor XIII deficiency needs several different assays: (i) clot solubility test, (ii) FXIII activity assay, (iii) FXIII antigen assay, (iv) inhibitor assay, and (v) molecular diagnostic and thromboelastography. Among them, the clot solubility test is widely used in developing countries for it being a simple, accessible, and cost‐effective screening method. Factor XIII activity assay was eventually undertaken due to abnormal clot solubility test results [1, 3].

Congenital factor XIII deficiency is autosomal recessive and affects all races and sexes equally [9]. In our case, the symptoms in the earliest days of life suggested the congenital form of the disease. Leukemia, severe liver disease, inflammatory bowel disease, disseminated intravascular coagulation, and systemic lupus erythematosus have all been linked to the disease. No such associations were found in our patient [10].

In terms of treatment of the condition, it should be tailored according to the needs of the patient. Often, the deficiency is treated with replacement. Two standard options are available: catridecacog, a recombinant factor XIII‐A subunit and plasma‐derived factor XIII. The plasma‐derived product contains both A and B subunits, making it suitable for all factor XIII‐deficient patients, regardless of whether their mutation affects subunit A or B. If factor XIII replacement products are not available, cryoprecipitate and FFP serve as viable options but with a risk of allergic reactions. We used FFP to manage the case with no allergies reported. It was due to the unavailability of other treatment options in our center and lack of interest of the patient's parents in expensive treatment. Prophylactic FFP was scheduled for every 1 month, as per literature advising replacement every 20–30 days but the patient followed up 2 months later [3]. It highlights the challenges in a low‐resource country where health cannot be maintained due to various constraints. Given the presentation of the baby with injuries in different parts of the body in different stages of healing suggested battered baby syndrome. It is essential to exclude this diagnosis in a country with a high prevalence.

Patients receiving proper treatments have good prognosis but recurrence occurs in 30% of central nervous system bleeding cases, with around 50% of these situations leading to death where intracranial hemorrhage stands as the primary cause [3, 5].

Diagnosis of such a condition is often overlooked in a low‐resource country. This is supported by a lack of diagnostic testing facilities, overwhelming patient volumes, and limited clinical exposure to specialized diagnostic protocols. Furthermore, because the initial umbilical cord stump bleeding stabilized following a standard blood transfusion, it masked the underlying pathology, appearing to not necessitate the need for further investigation and further evaluation. Only when the history of multiple petechiae and ecchymosis, delayed bleeding episodes following lip trauma, and normal hematological reports were obtained, did it give us a clue toward a hidden pathology. Thus, a high index of suspicion for factor XIII deficiency should be made in umbilical cord stump bleeding as suggested by Boon et al. [11].

## 6 Conclusion

This case highlights the importance of considering factor XIII deficiency in the absence of regular hematological test results. In a resource‐limited country, it is better to prevent the occurrence of complications than to treat it. FFP therapy has proven to be a good treatment option. Regular follow‐ups are a challenge in low‐resource countries and can help prevent the development of complications.

## Author Contributions

**Ramesh Yadav:** conceptualization. **Madhav Raj Karki:** writing – original draft. **Dikshya Pokhrel:** writing – original draft. **Aron Neupane:** writing – review and editing. **Kshitiz Parajuli:** writing – review and editing. **Hem Sagar Rimal:** writing – review and editing.

## Funding

The authors have nothing to report.

## Consent

Written informed consent was obtained from the patient's parents to publish this report in accordance with the journal's patient consent policy.

## Conflicts of Interest

The authors declare no conflicts of interest.

## Data Availability

Data will be provided by the corresponding author upon reasonable request.
